# Traumatic Right Iliac Fossa Abdominal Wall Hernia Containing Meckel's Diverticulum and Sigmoid Colon: A Case Report

**DOI:** 10.7759/cureus.74292

**Published:** 2024-11-23

**Authors:** Shaunak Tuvar, Ajay Nimbalkar, Ratnadip Sonawane

**Affiliations:** 1 General Surgery, Pimpri Chinchwad Municipal Corporation's (PCMC) Postgraduate Institute (PGI) Yashwantrao Chavan Memorial Hospital (YCMH), Pune, IND

**Keywords:** abdominal wall surgery, general trauma surgery, hernia, sigmoid colon, trauma

## Abstract

Traumatic abdominal wall hernia (TAWH) is a rare but serious condition resulting from blunt abdominal trauma, characterized by the herniation of bowel or abdominal organs through a disrupted musculature and fascia without skin penetration. This report describes a unique case of a 24-year-old man who sustained a high-velocity blunt abdominal injury from a motorcycle handlebar during a road traffic accident. The clinical presentation, diagnostic challenges, surgical intervention, and postoperative recovery are discussed to emphasize the importance of the early recognition and management of TAWH in trauma patients.

## Introduction

Traumatic abdominal wall hernia (TAWH) is a rare but critical diagnosis that is often overlooked in individuals who have experienced blunt abdominal trauma [1]. It is defined as the herniation of intra-abdominal contents through a defect in the abdominal wall musculature and fascia, typically resulting from significant trauma without skin penetration [2].

TAWH occurs due to a rapid increase in intra-abdominal pressure caused by a strong force applied over a small area of the abdomen. Tangential forces contribute to the rupture of the abdominal wall muscles and fascia, which leads to the subcutaneous herniation of abdominal viscera, particularly bowel loops, through the resulting defect. However, the superficial skin remains intact due to its elasticity [1].

Recognizing TAWH is vital, as overlooked cases can result in significant morbidity. Clinically apparent anterior TAWHs are often associated with a high incidence of additional injuries that require urgent laparotomy [3]. Despite advances in radiological investigations, maintaining a low threshold for early exploration is advisable to identify any concealed, undiagnosed injuries [4].

## Case presentation

A 24-year-old man presented to the emergency department in January 2023 after sustaining high-velocity abdominal trauma from a motorcycle accident. He fell from his motorcycle, and the handlebar struck his abdomen. He arrived at the hospital six hours after the trauma, reporting pain in the right iliac region and swelling in the right iliac fossa. He did not experience any vomiting or other symptoms. He was evaluated according to the advanced trauma life support (ATLS) protocol. Overall, he was hemodynamically stable with a Glasgow Coma Scale (GCS) score of 15/15.

**Figure 1:**
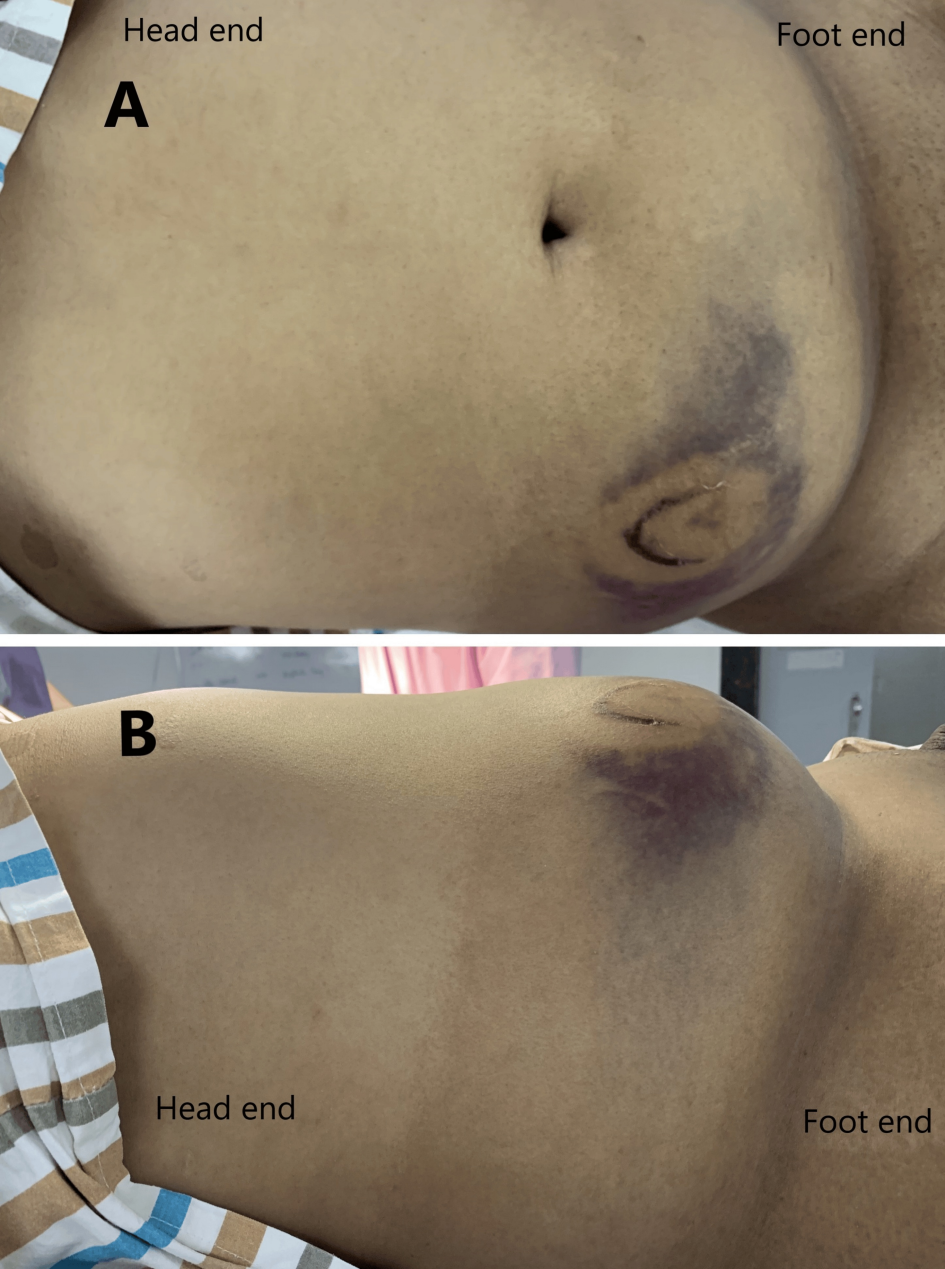
Traumatic abdominal wall hernia: (A) anterior view and (B) lateral view.

**Figure 2:**
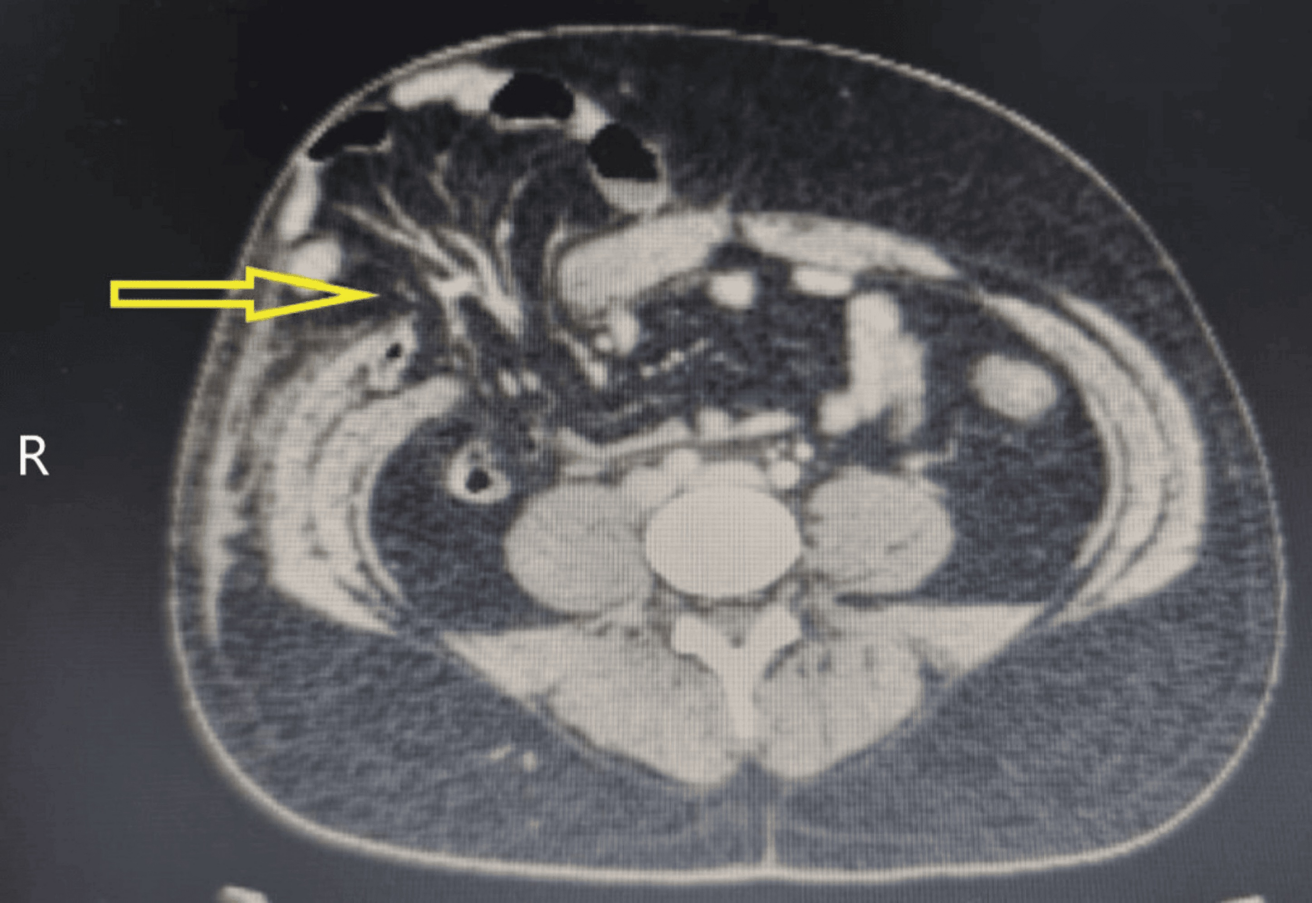
CT scan of the abdomen shows the hernia defect and the protruding contents of the hernia through the defect. CT: computed tomography

A thorough physical examination revealed a palpable swelling in the right iliac fossa, measuring approximately 5 cm × 4 cm. The swelling was firm, tender, and irreducible, and no cough impulse was elicited. Additionally, the skin over the swelling showed signs of ecchymosis (Figure 1). The rest of the abdomen was soft and non-tender, and the inguinoscrotal examination was normal. A contrast-enhanced computed tomography (CECT) scan of the abdomen showed an abdominal wall hernia containing omentum, bowel loops, and mesentery (Figure 2). There were no other injuries or fractures.

**Figure 3:**
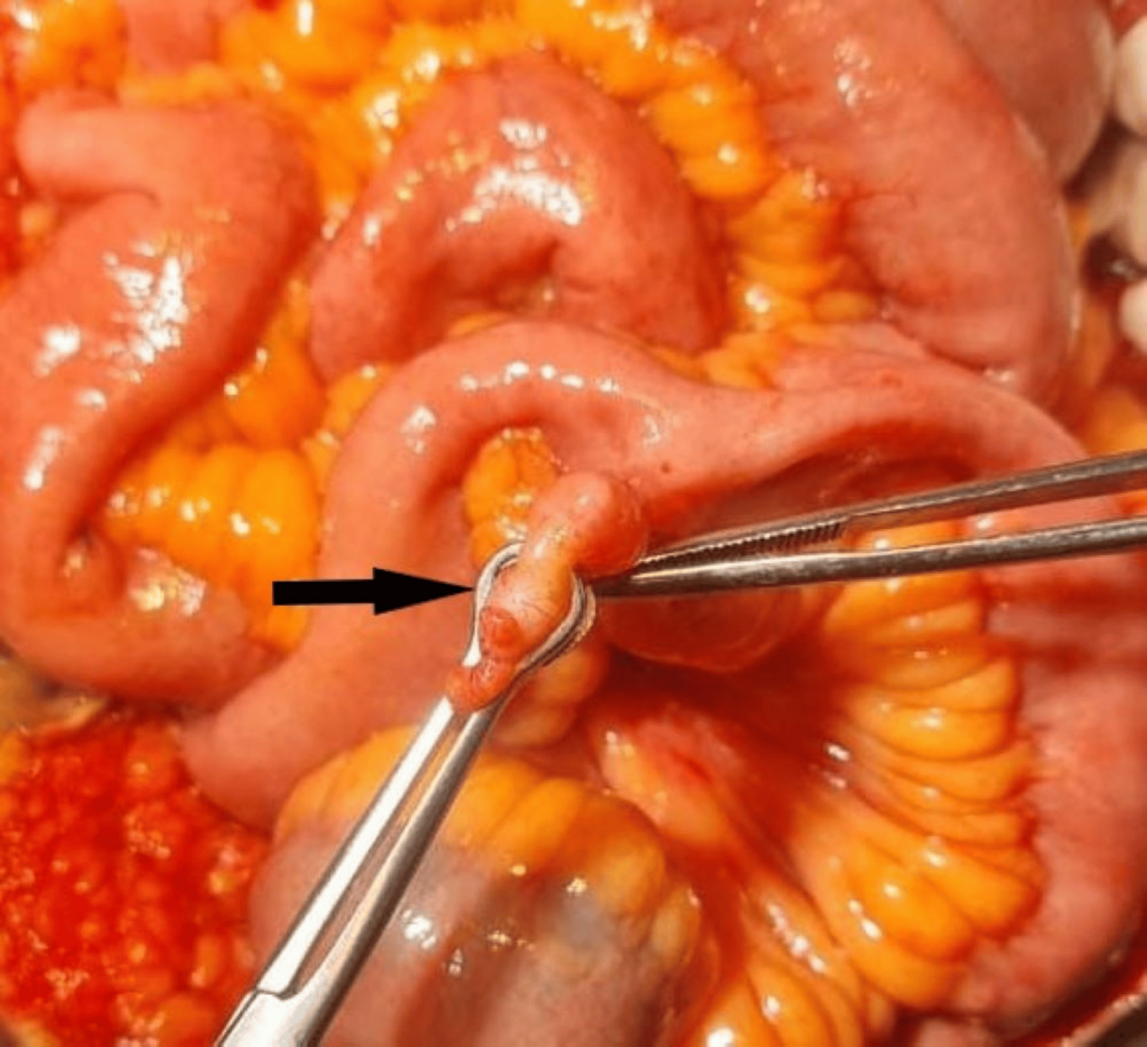
The herniated Meckel's diverticulum (arrow) along with the ileum.

**Figure 4:**
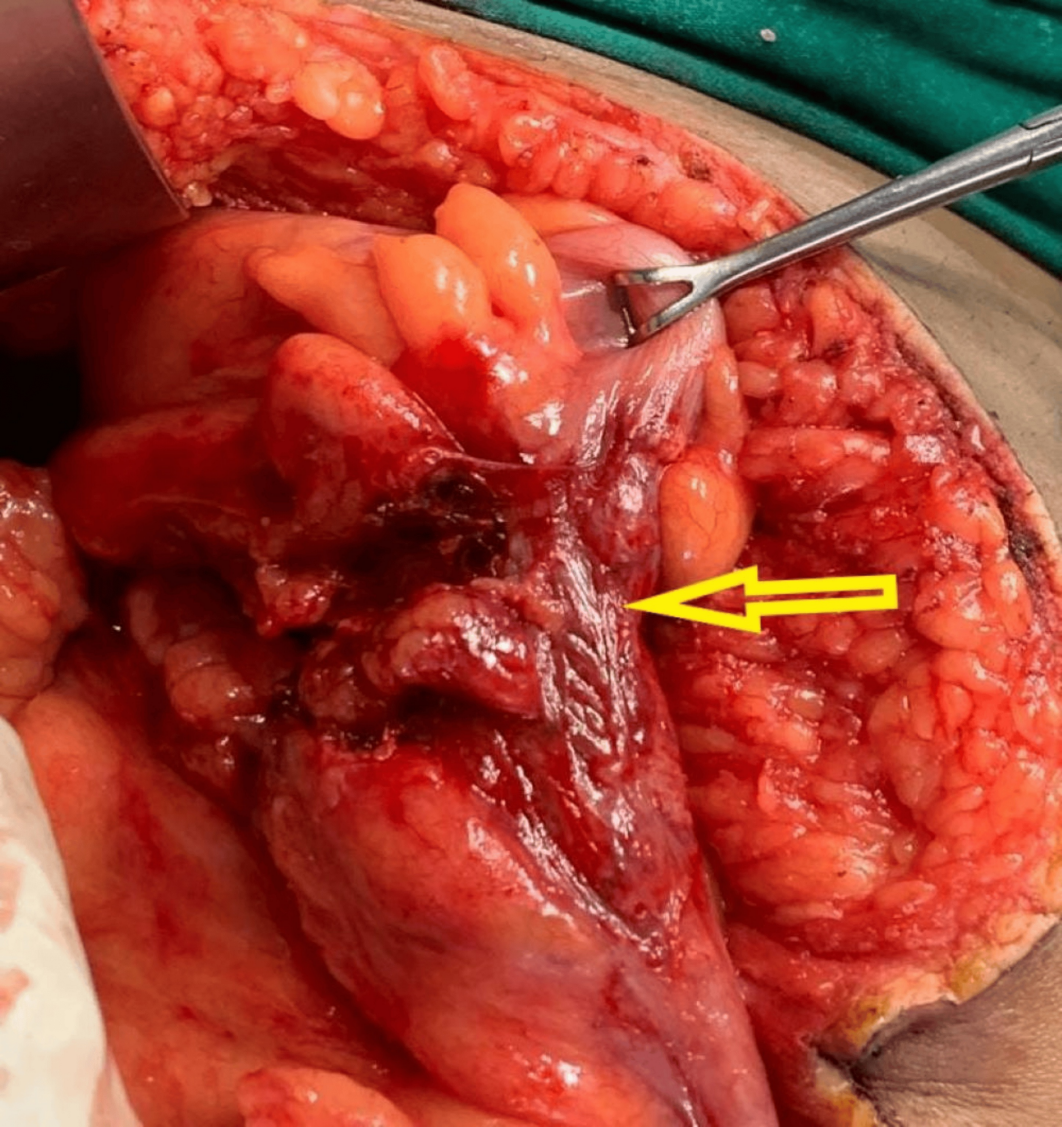
The ischemic sigmoid colon that was resected.

Based on clinical and radiological findings, the patient was taken for an emergency exploratory laparotomy. A midline incision was performed, which revealed herniated bowel loops and a defect measuring 5 cm × 5 cm in the right iliac fossa. The herniated segments of the ileum, along with Meckel's diverticulum, showed bluish-black discoloration, and the sigmoid colon appeared quite ischemic. The rest of the abdomen did not reveal any other injuries. After 15 minutes, the color of the small bowel and Meckel's diverticulum showed improvement (Figure 3). However, the herniated section of the sigmoid colon still appeared ischemic and displayed serosal damage in several areas (Figure 4). This was most likely due to blunt trauma causing bowel damage and some mesenteric injury.

The surgical resection of the damaged segment of the sigmoid colon was performed, followed by a single-layer colorectal anastomosis. Additionally, a wedge resection of Meckel's diverticulum was performed as there was no significant intra-abdominal contamination, and it was safe to proceed with it. A primary herniorrhaphy was performed to close the hernia defect, and the abdomen was thoroughly irrigated with warm saline before placing a drain. The histopathology of the sigmoid indicated significant edema, congestion, and hemorrhagic injury, with no signs of malignancy. The examination of the excised Meckel's diverticulum confirmed it to be a true diverticulum, comprising all three layers of the intestinal wall. There was no evidence of ectopic mucosa, diverticulitis, or malignancy.

The postoperative course was uneventful, and the patient was discharged on postoperative day 8 after full recovery. The patient was monitored at six months and one year, and there was no recurrence of hernia or any other issues during follow-up.

## Discussion

Traumatic abdominal wall hernias are rare, occurring in less than 1% of blunt trauma admissions. Diagnosing these hernias solely through physical examination can be challenging in an acute setting, with about 25% of cases often missed during initial evaluations [5].

A traumatic hernia occurs when an injury involves a concentrated force, such as that from a seat belt or a bicycle handlebar, impacting the abdomen. Although this force does not penetrate the abdominal cavity, it transmits energy throughout the abdominal wall. When this impact is combined with increased pressure, it can cause the muscle and fascia layers to shear, particularly at vulnerable anatomical sites such as the inguinal area or the lower abdomen near the rectus muscle. The skin's ability to stretch prevents it from tearing, which ultimately leads to the development of an abdominal wall hernia [6].

Lane et al. discuss management strategies for these hernias, emphasizing the need for comprehensive assessment and prompt surgical treatment. The treatment approach should be tailored to the patient's clinical condition and hemodynamic stability. For hernias resulting from low-energy injuries, repair can be performed through a direct incision above the hernia site. In contrast, hernias caused by high-energy trauma require an exploratory laparotomy via a midline incision due to the increased risk of other missed intra-abdominal injuries [7].

Enhanced safety in surgical procedures and anesthesia, coupled with the improved management of potential complications, supports a careful preference for the resection of Meckel's diverticulum. The existing evidence leans toward resection; however, factors such as patient characteristics and the primary condition will affect this choice, as highlighted in a recent systematic review that included 42 relevant studies on the outcomes of managing asymptomatic Meckel's diverticulum [8].

Tension-free mesh repair for hernias is effective in reducing recurrence rates. However, if early exploration is needed, related injuries may increase the risk of infections associated with the mesh. Specifically, injuries to hollow organs that lead to abdominal contamination are seen as contraindications for using mesh in these repairs [9].

Rosen et al. (2013) noted that biological mesh might be a suitable option in cases with intra-abdominal contamination, despite its less favorable long-term durability [10]. In cases where patients present with a large defect (with a low risk of strangulation) and no intra-abdominal injuries, a delayed elective repair may be appropriate. In this scenario, the surrounding tissue remains healthy, allowing for the use of mesh. However, if left untreated, the defect might enlarge, and muscle atrophy could complicate primary closure [11].

In our case, the hernia was strangulated, necessitating immediate surgical exploration and primary repair.

## Conclusions

Traumatic abdominal wall hernia is a rare but potentially life-threatening condition that demands careful clinical assessment and prompt intervention. This case emphasizes the importance for clinicians to maintain a high level of suspicion for TAWH in patients who have experienced blunt abdominal trauma. Early recognition and appropriate surgical management are essential to optimize patient outcomes and prevent complications.

## References

[1] Pothiawala S, Balasubramaniam S, Taib M, Bhagvan S (2022). Traumatic abdominal wall hernia: a rare and often missed diagnosis in blunt trauma. World J Emerg Med.

[2] Singal R, Dalal U, Dalal AK (2011). Traumatic anterior abdominal wall hernia: a report of three rare cases. J Emerg Trauma Shock.

[3] Netto FA, Hamilton P, Rizoli SB, Nascimento B Jr, Brenneman FD, Tien H, Tremblay LN (2006). Traumatic abdominal wall hernia: epidemiology and clinical implications. J Trauma.

[4] Pathak D, Mukherjee R, Das P, Pathak D, Gangopadhyay A, Das S (2016). Traumatic abdominal wall hernia with concealed colonic perforation. Ann R Coll Surg Engl.

[5] Chow KL, Omi EC, Santaniello J (2020). Traumatic abdominal wall hernias: a single-center case series of surgical management. Trauma Surg Acute Care Open.

[6] van Bemmel AJ, van Marle AG, Schlejen PM, Schmitz RF (2011). Handlebar hernia: a case report and literature review on traumatic abdominal wall hernia in children. Hernia.

[7] Lane CT, Cohen AJ, Cinat ME (2003). Management of traumatic abdominal wall hernia. Am Surg.

[8] Yagnik VD, Garg P, Dawka S (2024). Should an incidental Meckel diverticulum be resected? A systematic review. Clin Exp Gastroenterol.

[9] Liasis L, Tierris I, Lazarioti F, Clark CC, Papaconstantinou HT (2013). Traumatic abdominal wall hernia: is the treatment strategy a real problem?. J Trauma Acute Care Surg.

[10] Rosen MJ, Krpata DM, Ermlich B, Blatnik JA (2013). A 5-year clinical experience with single-staged repairs of infected and contaminated abdominal wall defects utilizing biologic mesh. Ann Surg.

[11] Yadav S, Jain SK, Arora JK (2013). Traumatic abdominal wall hernia: delayed repair: advantageous or taxing. Int J Surg Case Rep.

